# Supplementary material for: CoA‐dependent activation of mitochondrial acyl carrier protein links four neurodegenerative diseases
Source: EMBO Mol Med. 2019 Nov 7;11(12):e10488. doi: 10.15252/emmm.201910488 (PMC6895606; doi:10.15252/emmm.201910488)
Supplement: Supplementary file 1 — Appendix [file EMMM-11-e10488-s001.pdf]

CoA-dependent activation of mitochondrial acyl carrier protein links four neurodegenerative diseases

Lambrechts et al, 2019

APPENDIX FIGURES, LEGENDS and TABLES

Table of contents:

- **Appendix Figure S1:** Detection of lipoylated proteins
- **Appendix Figure S2:** PCR analysis of outcrossed stocks to generate *bona fide* RNAi lines for *dPANK/fbl* and *dPPCDC/ppcdc*.
- **Appendix Figure S3:** Crossing scheme explaining reduced viability in *dPANK/fbl-RNAi* flies
- **Appendix Figure S4:** Downregulation of *dPANK/fbl* and *mtACP* with RNAi constructs using the wing specific driver *MS1096-GAL4* in developing larval wing discs
- **Appendix Figure S5:** Controls for the specificity of the effect of the *PDK-RNAi* and the *SIRT4-RNAi* lines
- **Appendix Figure S6:** Control wings, testing non-relevant overexpression- or RNAi constructs in combination with *dPPCDC/ppcdc-RNAi*
- **Appendix Figure S7:** Verification of doxycycline inducibility of the RNAi hairpin in HEK293T cells
- **Appendix Figure S8:** Verification of doxycycline inducibility of the RNAi hairpin in SH-SY-5Y cells
- **Appendix Figure S9:** qPCR data demonstrates reduced mRNA expression of PANK2 in inducible lines PANK2.1 and 2.3 in HEK293T cells, while PANK1, 3 and 4 expression remain unaffected
- **Appendix Figure S10:** Origene antibody # TA501321 detects hPANK2 migrating at 48 kD
- **Appendix Figure S11:** Measurements of CoA levels
- **Appendix:** References associated with Appendix figures
- **Appendix Fly Genetics:** Fly stocks and genotypes
- **Appendix Table 1:** Vectors and Cell Lines
- **Appendix Table 2:** List of qPCR Primers
- **Appendix Table 3:** Antibody List

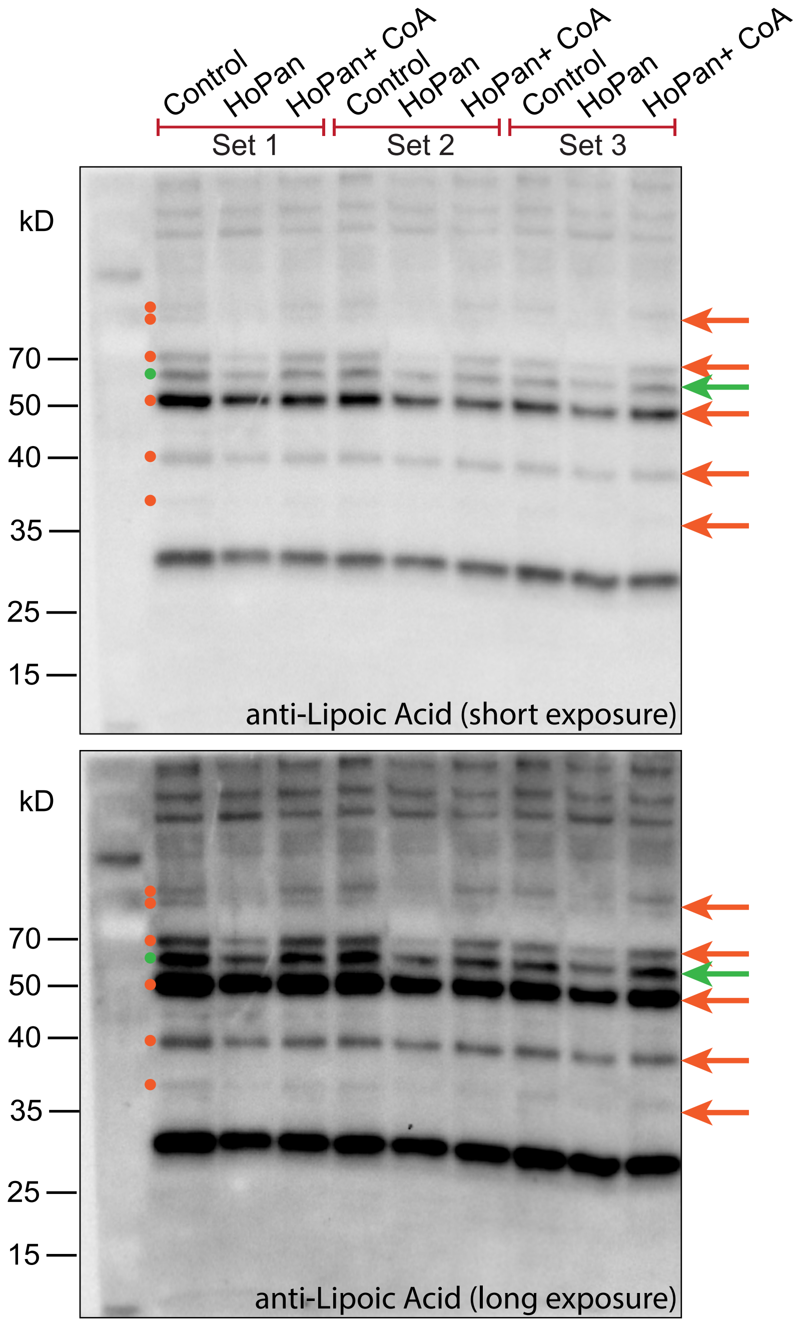

Appendix Figure S1: Detection of lipoylated proteins.

Western blot analysis showing several lipoylated proteins under control conditions, after HoPan treatment and after HoPan + CoA treatment. S2 cells were treated with HoPan or HoPan + CoA for 4 days; non-treated cells were used as control. 3 separate experiments are shown (set 1-3). The green arrow/dot points towards lipoylated PDH-E2, the red arrows/dots indicate other lipoylated proteins. Upon HoPan treatment, lipoylation of these bands decreases, which can be rescued upon HoPAN + CoA treatment. Short exposure (top) and long exposure (bottom) are provided to clearly distinguish strong and weak lipoylated proteins.

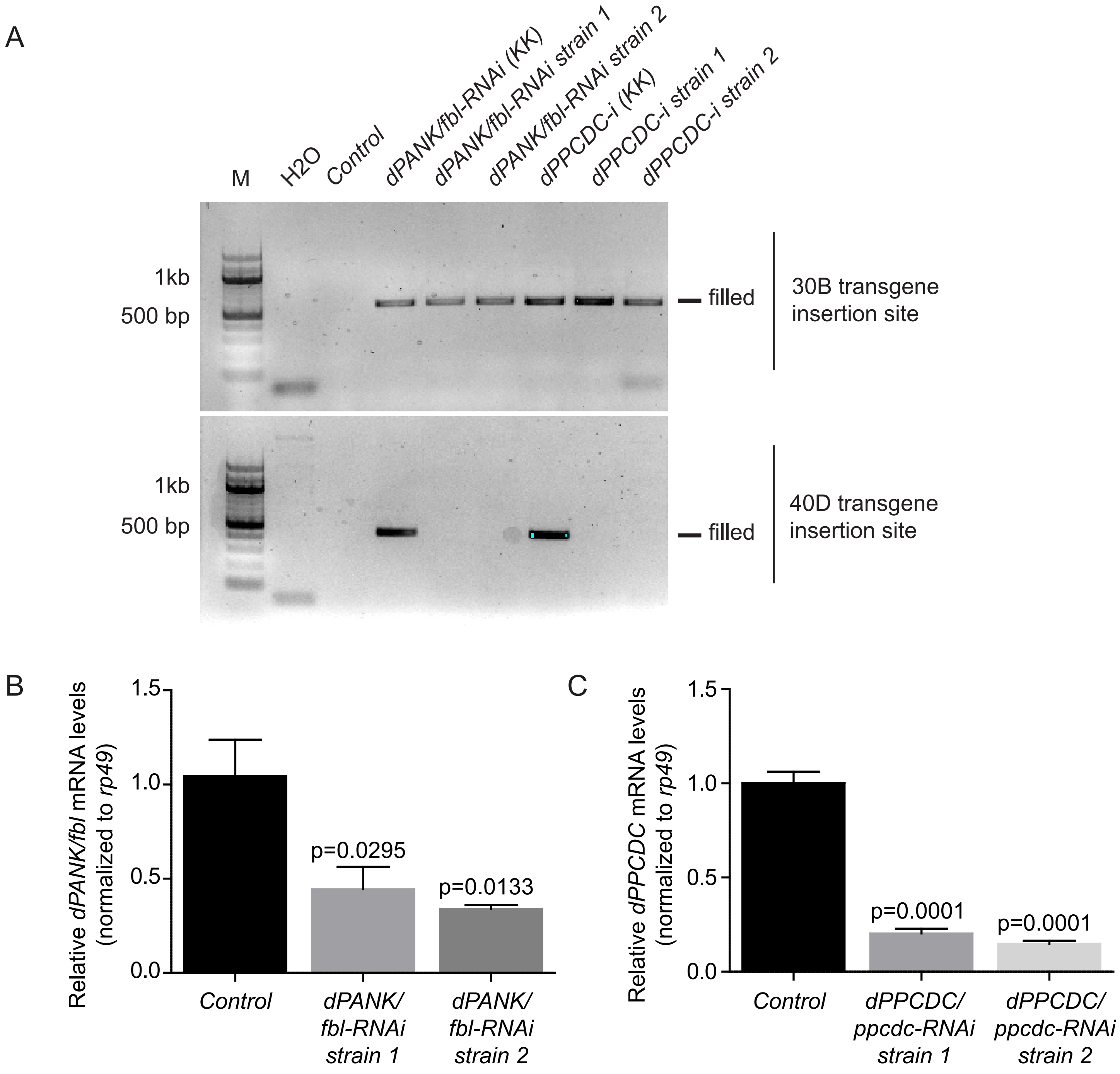

A
M
H2O
Control
dPANK/fbl-RNAi (KK)
dPANK/fbl-RNAi strain 1
dPANK/fbl-RNAi strain 2
dPPCDC-i (KK)
dPPCDC-i strain 1
dPPCDC-i strain 2
1kb
500 bp
filled
30B transgene insertion site
1kb
500 bp
filled
40D transgene insertion site
B
Relative dPANK/fbl mRNA levels (normalized to rp49)
1.5
1.0
0.5
0.0
p=0.0295
p=0.0133
Control
dPANK/ fbl-RNAi strain 1
dPANK/ fbl-RNAi strain 2
C
Relative dPPCDC mRNA levels (normalized to rp49)
1.5
1.0
0.5
0.0
p=0.0001
p=0.0001
Control
dPPCDC/ ppcdc-RNAi strain 1
dPPCDC/ ppcdc-RNAi strain 2

Appendix Figure S2: PCR analysis of outcrossed stocks to generate *bona fide* RNAi lines for *dPANK/fbl* and *dPPCDC/ppcdc*.

Original *Drosophila* VDRC lines containing *RNAi* constructs directed against *dPANK/fbl* and *dPPCDC/ppcdc (*here referred to as KK lines*)* contained in addition to the functional transgene insertion site at position 30B in the genome an additional transgene insertion site at position 40D, causing, when overexpressed, a wing phenotype not related to the *RNAi* construct (Green et al., 2014; Vissers et al., 2016). These unwanted extra insertion sites were removed by outcrossing and “cleaned-up” fly stocks were generated and verified by PCR. The cleaned-up and thereby *bona fide Drosophila* strains were used for all experiments.

(A) From the original *Drosophila KK* lines. the additional 40D transgene insertion site was removed and the *bona fide* strains were controlled by PCR for the absence of this additional 40D transgene insertion and the presence of the functional 30B transgene insertion site harbouring the specific RNAi constructs. The PCR was performed as previously described (Vissers et al., 2016)and run on a 2% agarose gel. M: 100bp marker; H_2O: water-only control; control wild type stock used for recombination, not containing either (30B/40D) element; *dPANK/fbl-RNAi-KK*: Original VDRC KK *fbl-RNAi* line, containing both 30B and 40D elements; *dPANK /fbl-RNAi-strain 1*: “cleaned-up” recombinant line 1 containing only the 30B *dPANK/fbl-RNAi* construct; *dPANK /fbl-RNAi-strain 2*: “cleaned-up” recombinant line 2 containing only the 30B *dPANK/fbl-RNAi* construct; *dPPCDC/ppcdc-RNAi-KK*: Original VDRC KK *dPPCDC/ppcdc-RNAi* line, containing both 30B and 40D elements; *PPCDC-RNAi-strain 1* and *strain 1*: “cleaned-up” recombinants 1 and 2 containing only the 30B *PPCDC-RNAi* construct.

(B) Indicated RNAi constructs are ubiquitously expressed using an *Actin-GAL4* driver. Efficiency of *dPANK/fbl* downregulation by *RNAi* was verified for the two “cleaned-up” lines *dPANK/fbl*-RNAi-*strain 1* and *dPANK/fbl*-RNAi-*strain 2* by qPCR.

(C) Efficiency of *dPPCDC/ppcdc* downregulation by *RNAi* was verified for two of the “cleaned-up” lines *dPPCDC/ppcdc-RNAi-strain 1 and dPPCDC/ppcdc-RNAi-strain 2* by qPCR.

For B and C mean ±SD is given. One-tailed, unpaired Student t-tests were performed to asses statistical significance for the indicated subsets. n=3 for all samples.

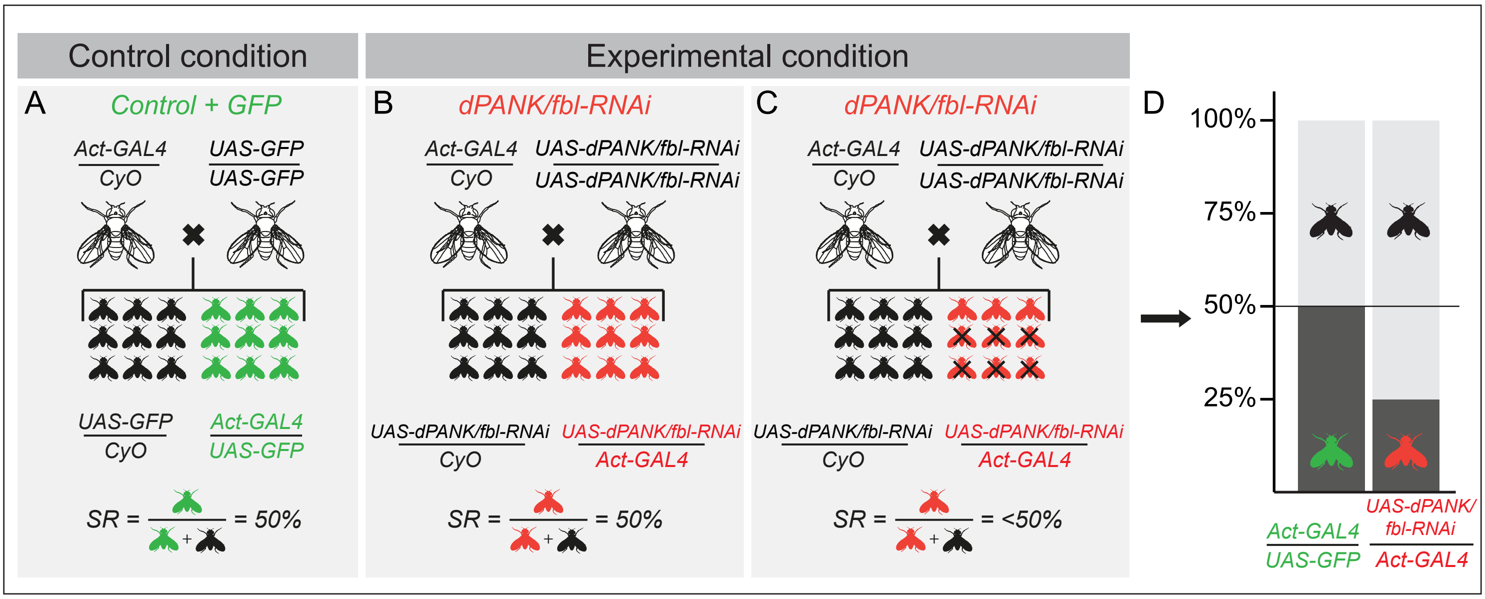

Appendix Figure S3: Crossing scheme explaining reduced viability in *dPANK/fbl-RNAi* flies

(A-D) Viability of *dPANK/fbl*-RNAi expressing flies was determined and compared to control flies using the following assay: The *Act-GAL4* driver was used to express *UAS-dPANK/fbl* RNAi ubiquitously in all cells of the flies. As a control the *Act-GAL4* driver was used to ubiquitously express a non-relevant construct (green fluorescent protein (GFP)). The genotype of the flies used for the crosses is indicated. (A) For the controls, *Act-GAL4*/*CyO* flies were crossed with a fly strain homozygous for *UAS-GFP* (*UAS-GFP/UAS-GFP*). *CyO* is a dominant marker on an otherwise wildtype chromosome and allows following that chromosome. The number of adult progeny of this cross, hatching after pupal development, was determined and thereby the survival rate (SR) per genotype was calculated. Based on Mendelian genetics, in the control cross, 50% of the progeny will have an *Act-GAL4/UAS-GFP* genotype (expressing the GFP construct, indicated as green flies) and 50% will have a *CyO*/*UAS-GFP* genotype (not expressing the construct, indicated as black flies). Because expression of the GFP construct is not detrimental, the survival rate of the *Act-GAL4/UAS-GFP* genotype is 50% in the control cross.

(B/C) To determine the survival rate of *dPANK/fbl-RNAi* expressing flies, the following cross was performed: *Act-GAL4/CyO* expressing flies were crossed with a fly strain homozygous for *UAS-dPANK/fbl-RNAi*. The amount of surviving flies was determined. (B) In the situation that *UAS-dPANK/fbl-RNAi* expression is not detrimental, the progeny will consist of 50% *UAS-dPANK/fbl-RNAi/CyO* (with normal levels of dPANK/Fbl protein because there is no expression of the RNAi construct, indicated as black flies) and 50% will be *UAS-dPANK/fbl-RNAi/Act-GAL4* (with decreased levels of *dPANK/fbl*, because *Act-GAL4* drives the expression of the RNAi construct, indicated as red flies). (C) In the situation that expression of *UAS-dPANK/fbl-RNAi* is detrimental and leads to reduced viability, the viability of *UAS-dPANK/fbl-RNAi/Act-GAL4* flies will be less than 50% of the total.

(D) Visualizes a possible outcome caused by a decreased viability of *UAS-dPANK/fbl-RNAi* expressing flies compared to GFP expressing flies, both driven by the same *Act-GAL4* driver.

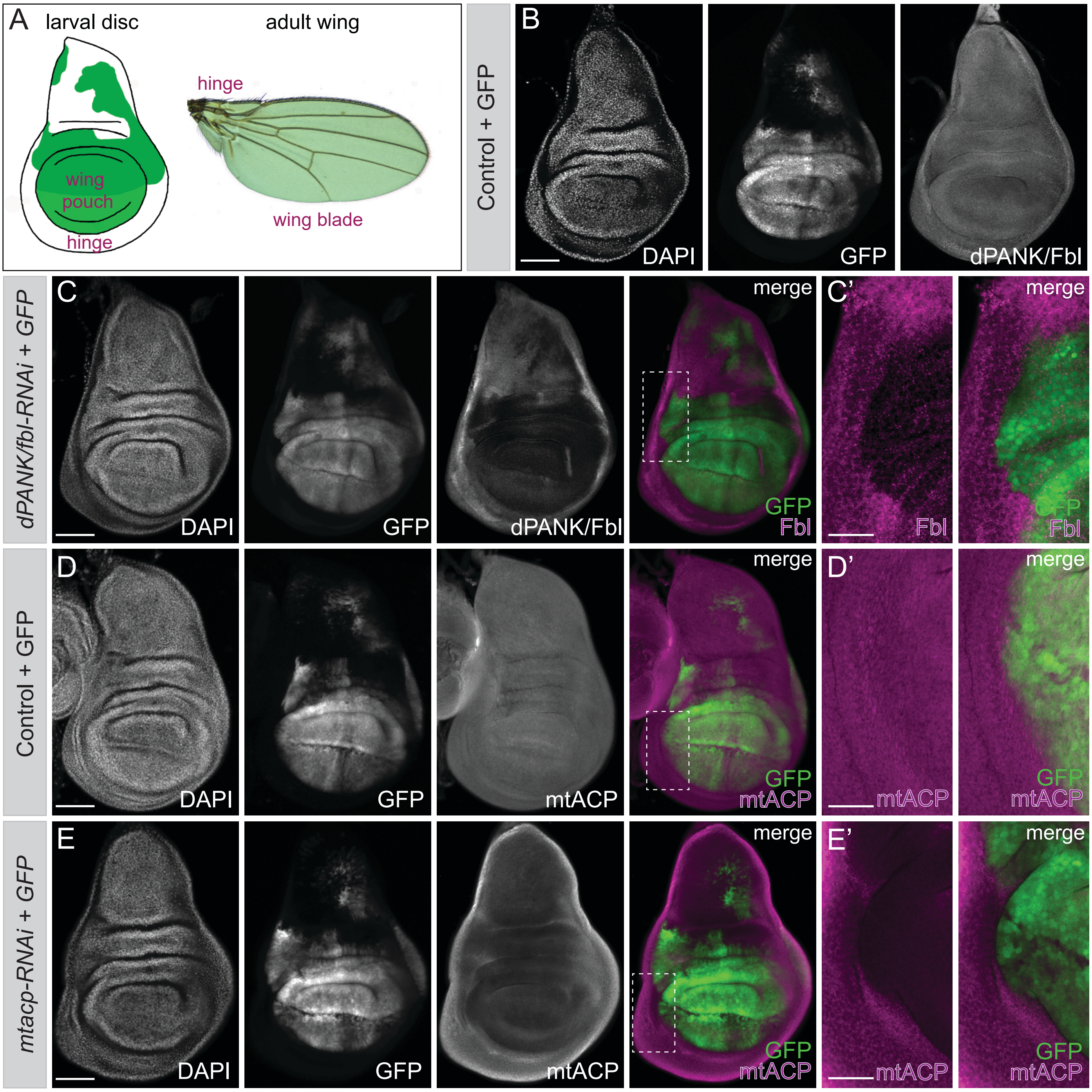
A
larval disc
adult wing
hinge
wing
pouch
hinge
wing blade
Control + GFP
B
DAPI
GFP
dPANK/Fbl
dPANK/fbl-RNAi + GFP
C
DAPI
GFP
dPANK/Fbl
merge
GFP
Fbl
C'
Fbl
merge
GFP
Fbl
Control + GFP
D
DAPI
GFP
mtACP
merge
GFP
mtACP
D'
mtACP
merge
GFP
mtACP
mtacp-RNAi + GFP
E
DAPI
GFP
mtACP
merge
GFP
mtACP
E'
mtACP
merge
GFP
mtACP

Appendix Figure S4: Downregulation of *dPANK/fbl* and *mtACP* with RNAi constructs using the wing specific driver *MS1096-GAL4* in developing larval wing discs.

(A) Schematic representation of the expression pattern of the wing driver (*MS1096-GAL4*) (marked in green) in the third instar larval wing disc and the adult wing resulting from it. In contrast to the ubiquitously expressed *Actin-Gal4* driver (*Act-GAL4*), the wing specific driver *MS1096-GAL4* only targets expression of the indicated genes in the larval wing pouch, which gives rise to the wing in the adult fly. Defects induced during development of the larval wing disc by the expression of various RNAis will lead to abnormalities in the adult wing, which are straightforward to score (see Figure 5).

(B) Validation of the wing driver *MS1096-GAL4* by expressing GFP under control of the driver. The wing pouch area tested positive for GFP. These wing discs were also tested for dPANK/Fbl (visualized by staining with an dPANK/Fbl antibody), which is visible in the entire wing disc in B. The nuclear marker DAPI was used to visualize the overall structure of the wing disc.

(C-E) *Drosophila* genetics allowed co-expression of GFP (to highlight the area) together with the *dPANK/fbl- or mtacp-RNAi* constructs in the wing pouch. The downregulation of the gene products (dPANK/Fbl or mtACP) in the larval wing disc was determined using available antibodies (in grey/magenta). DAPI was used to visualize the structure of the complete wing disc (grey), while GFP marks the area in which the RNAi constructs are expressed (grey/green). In (C/C') levels of dPANK/Fbl protein are decreased in the wing pouch, in (E/E') levels of mtACP. This demonstrates the specificity of the wing pouch driver, the effectivity of RNAi constructs, and the specificity of the used antibodies. The boxed areas (C-E) are enlarged in the adjacent images to show the decrease/absence of the indicated proteins in the GFP positive areas in more detail. *dPPCDC/ppcdc* RNAi *,* for which no antibody was available, was validated by qPCR in Appendix Figure S2.

Scale bars: C/D/E = 50 µm, C'/D'/E' = 20 µm.

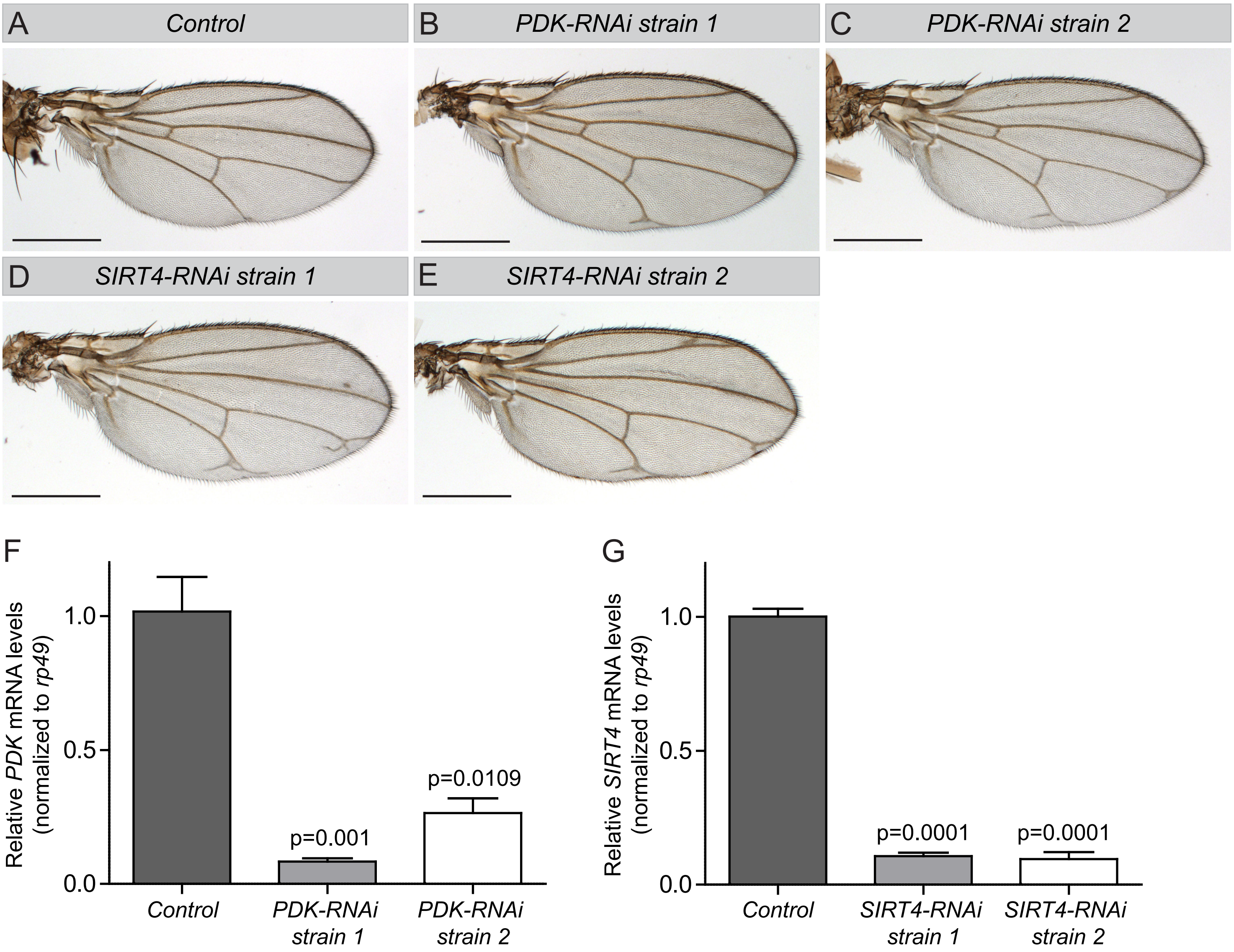

Appendix Figure S5: Controls for the specificity of the effect of the *PDK-RNAi* and the *SIRT4-RNAi* lines

Downregulation of PDK or SIRT4 by expression of *PDK-RNAi* or *SIRT4-RNAi* respectively did not result in a wing blister phenotype. For each gene, two independent RNAi strains were chosen.

(A) control adult wing, (B, C) for *PDK-RNAi* and (D-E) for *SIRT4-RNAi*.

Scale bar = 500μm (A-E)

(F) Efficiency of *PDK* downregulation by *RNAi* (strain 1 & strain 2) was verified by qPCR.

(G) Efficiency of *SIRT4* downregulation by *RNAi* (strain 1 & strain 2) was verified by qPCR.

For F and G mean ±SD is given. One-tailed, unpaired Student t-tests were performed to compare indicated subsets. n=3 for all samples.

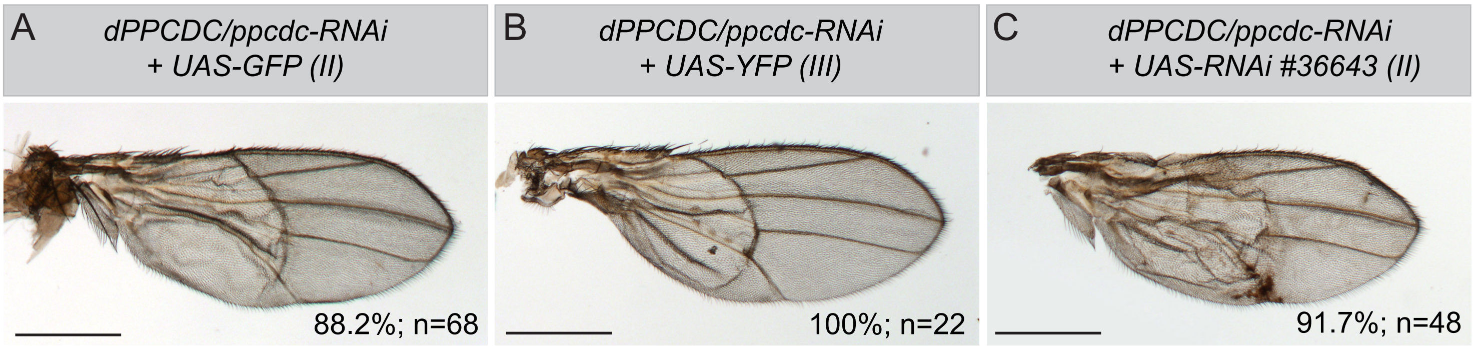

Appendix Figure S6: Control wings, testing non-relevant overexpression- or RNAi constructs in combination with *dPPCDC/ppcdc-RNAi*

(A-C) To test if other non-relevant overexpression- or RNAi-lines could influence the *dPPCDC/ppcdc-RNAi* knockdown phenotype in the wing, we co-expressed *dPPCDC/ppcdc-RNAi* with GFP (A), YFP (B) and a randomly chosen *TRiP-RNAi #36643* (C). No rescue of the blistering phenotype was observed for any of the combinations.

Scale bars = 500µm

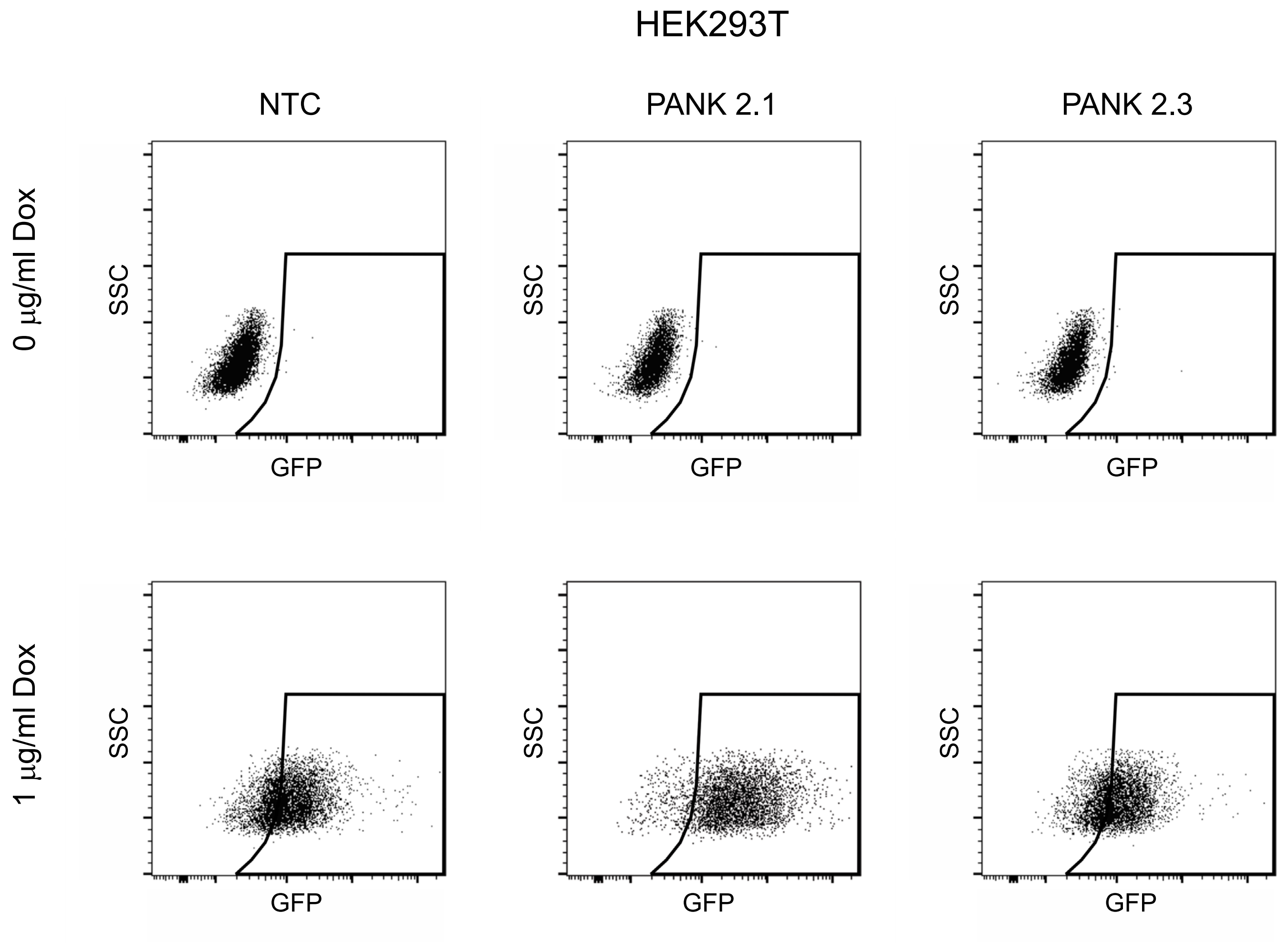

Appendix Figure S7: Verification of doxycycline inducibility of the RNAi hairpin in HEK293T cells

HEK293T cells were transduced with a lentivirus construct targeting human PANK2 (only hairpins 2.1 and 2.3 are shown) or a Non-Targeted Control (NTC) construct and subsequently selected with 0.6 µg/ml puromycin. After 1 week, Doxycycline was added for 3 days to induce the microRNA coupled with a TurboGFP cassette, to enable selection by FACS sorting and the cells were subsequently analysed on an LSR-II flow cytometer (only data from concentration of 1 µg/ml is shown). Induction with doxycycline shows a clear induction of TurboGFP expression and hence the coupled hPANK2-RNAi expression to downregulate hPANK2.

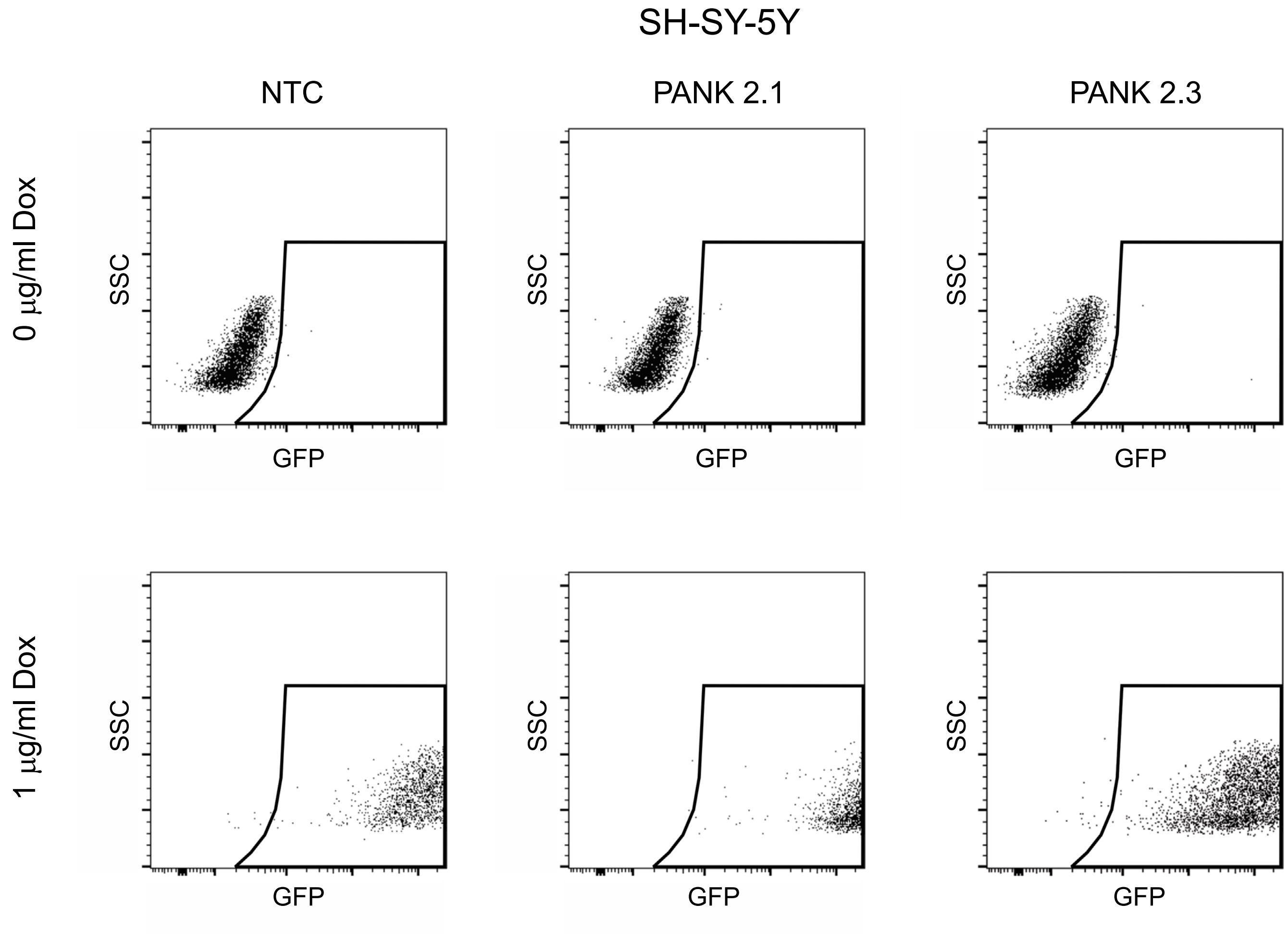

Appendix Figure S8: Verification of doxycycline inducibility of the RNAi hairpin in SH-SY-5Y cells

SH-SY5Y cells were transduced with a lentivirus construct targeting human PANK2 (only hairpins 2.1 and 2.3 are shown) or a Non-Targeted Control (NTC) construct and subsequently selected with 0.2 µg/ml puromycin. After 1 week, Doxycycline was added for 3 days to induce the microRNA coupled with a TurboGFP cassette to enable selection by FACS sorting and the cells were subsequently analysed on an LSR-II flow cytometer (only data from concentration of 1 µg/ml is shown). Induction with doxycycline shows a clear induction of TurboGFP expression and hence the coupled hPANK2-RNAi expression to downregulate hPANK2.

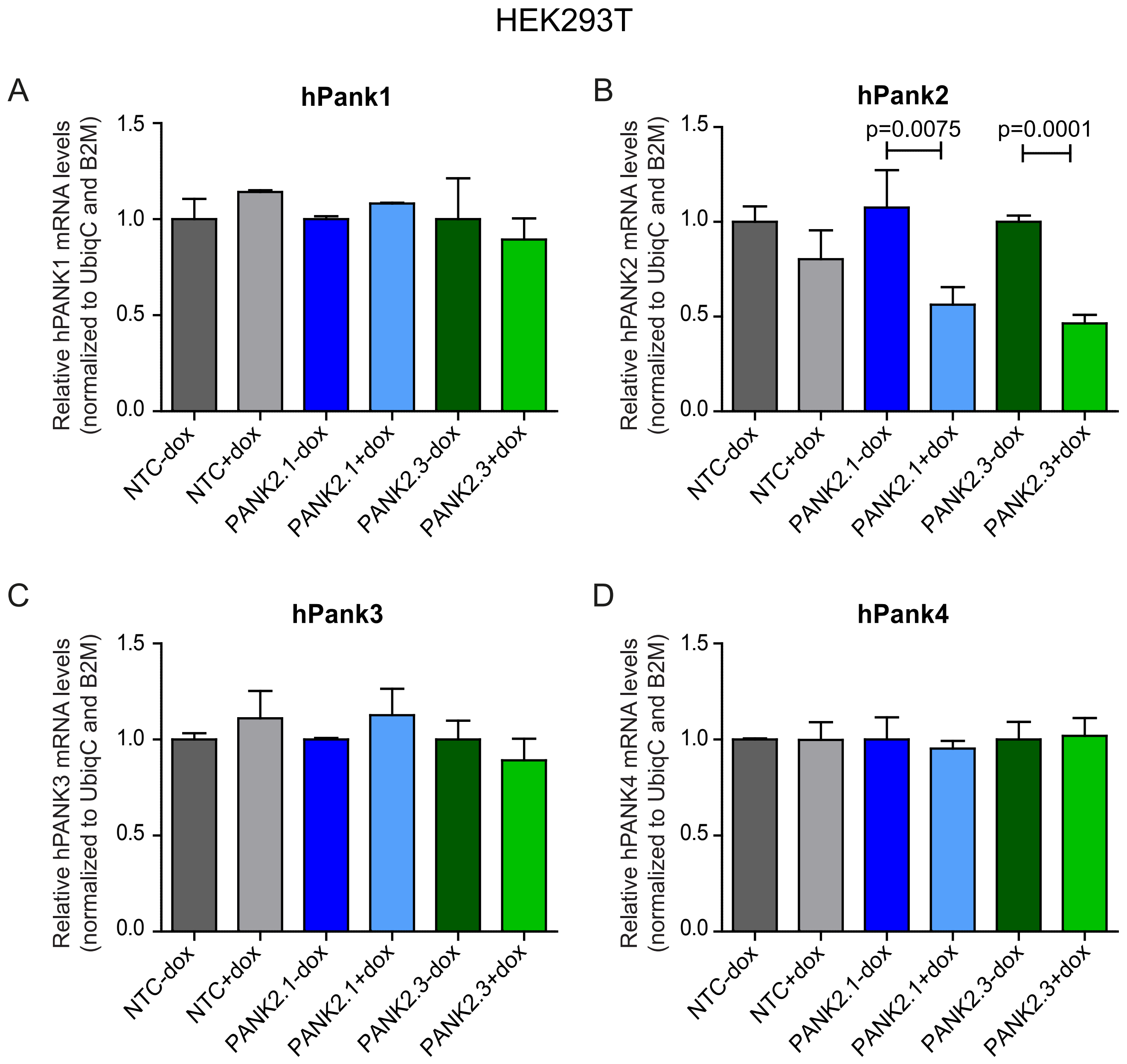

Appendix Figure S9: qPCR data demonstrates reduced mRNA expression of PANK2 in inducible lines PANK2.1 and 2.3 in HEK293T cells, while PANK1, 3 and 4 expression remain unaffected

qPCR on non-treated control cells (NTC) and doxycycline inducible PANK2 HEK293T knockout lines, PANK2.1 and 2.3, with and without doxycycline.

(A) mRNA expression levels of PANK1 are unaffected in the inducible PANK2 knockout lines after doxycycline treatment.

(B) mRNA expression levels of PANK2 show a decrease in the inducible PANK2 knockout lines after doxycycline treatment.

(C) mRNA expression levels of PANK3 are unaffected in the inducible PANK2 knockout lines after doxycycline treatment.

(D) mRNA expression levels of PANK4 are unaffected in the inducible PANK2 knockout lines after doxycycline treatment.

For A-D mean ±SD is given. One-tailed, unpaired Student t-tests were performed to compare indicated subsets. n=3 for all samples.

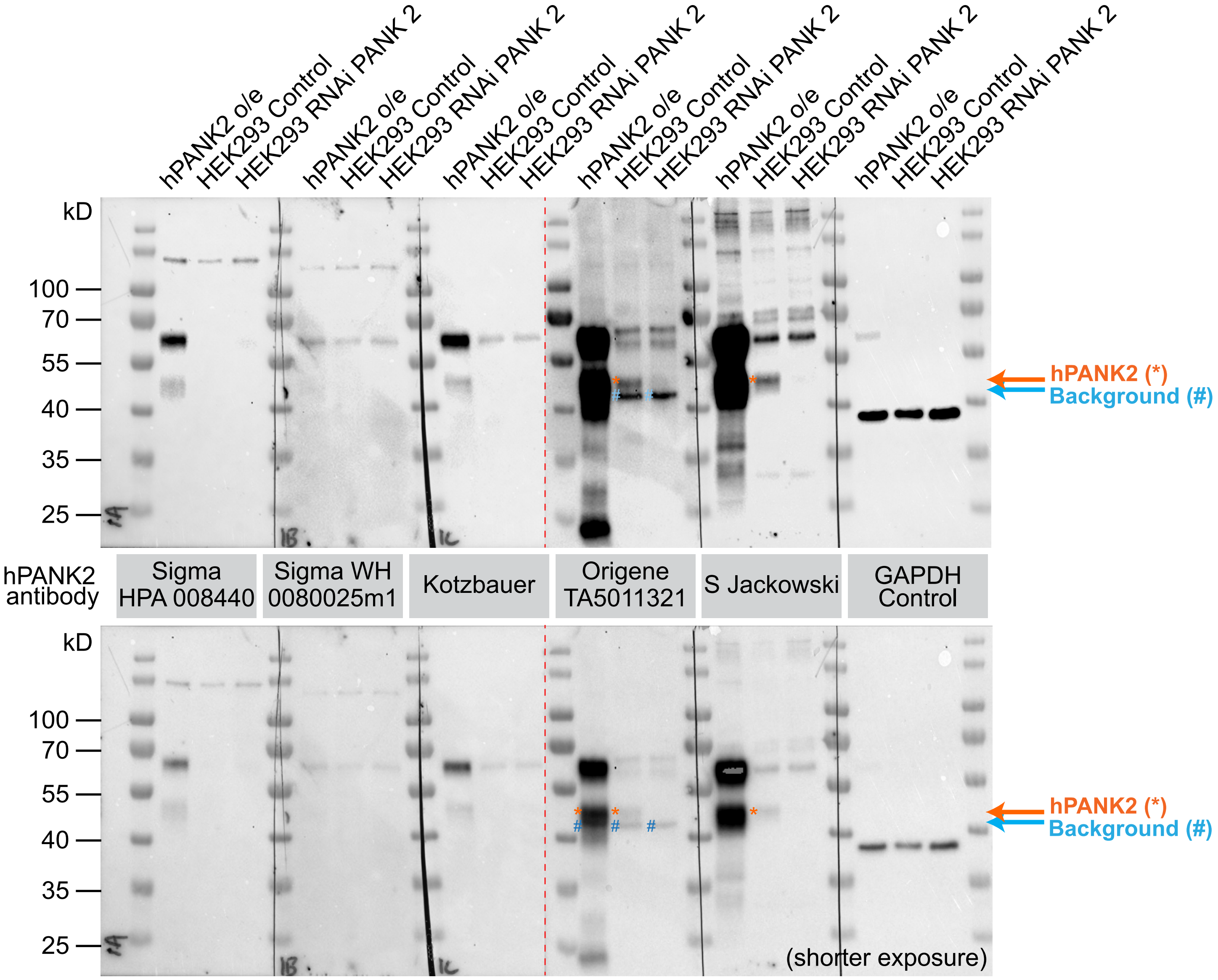

Appendix Figure S10: Origene antibody # TA501321 detects hPANK2 migrating at 48 kDa

Lysates were generated to determine the specificity of various commercially available antibodies. For this three different lysates were used: lysate 1: HEK293 cells in which human PANK2 was overexpressed (indicated as hPANK2 o/e); lysate 2: control HEK293 cells which endogenously express human PANK2; lysate 3: HEK293 cells in which human PANK2 is downregulated by doxycycline inducible hPANK2 RNAi expression.

All three lysates were used for Western blot analysis using 5 antibodies: 1: commercially available Sigma HPA 008440; 2: commercially available Sigma HPA 008025m1; 3: human PANK2 antibody obtained as a gift from Prof. Kotzbauer; 4: commercially available antibody Origene TA 501321; 5: human PANK2 antibody obtained as a gift from Prof. S. Jackowski. In this experiment, the lane 1, containing overexpression of human PANK2 serves as the positive control and lane 3, containing samples in which human PANK2 is downregulated by RNAi serves as the negative control. The two Sigma antibodies and the antibody from Prof. Kotzbauer do recognize human PANK2 when it is overexpressed, however, they do not detect endogenous human PANK2. The antibody from Origene and the antibody from Prof. Jackowski do recognize endogenous human PANK2 and a specific signal is obtained migrating at the same mobility (around 48 kDa, (Kotzbauer et al., 2005) as the protein detected by overexpression of human PANK2. This specific band, indicated by a *, is absent in the PANK2 downregulated cells, demonstrating both the effectivity of the human PANK2 downregulation and the specificity of the antibodies for endogenous human PANK2. In addition to the specific human PANK2 signal, the antibody from Origene also detects a background band, which is not detected by the antibody from Prof. Jackowski. This band is therefore considered a background band, and is indicated with a #. Two exposure times are presented, a long and short exposure time and GAPDH was used as a loading control.

For the human PANK2 Western blot in Figure 7, the antibody from Origene was used.

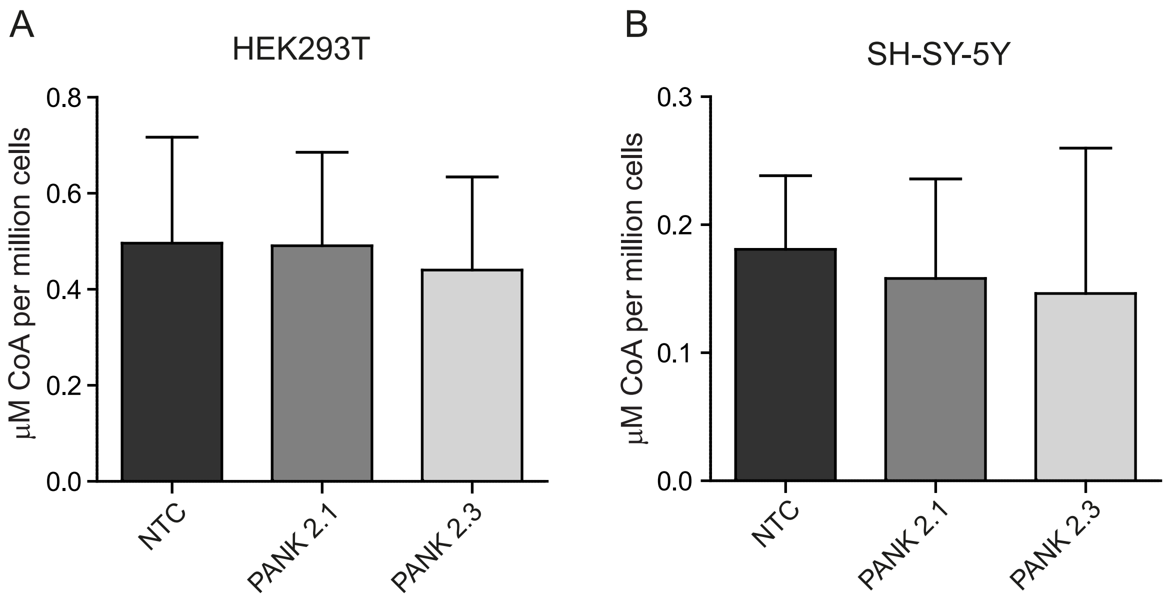

Appendix Figure S11: Measurements of CoA levels.

CoA levels were measured using HPLC as described in (Srinivasan et al., 2015) in the indicated clones of the HEK293T cells (A) and SH-SY-5Y cells (B) after 10 days of RNAi treatment. HEK293T and SH-SY-5Y cells were cultured in Vitamin B5-deprived medium. n=3 for all samples. μM CoA per million cells is shown.

References associated with Appendix Figures

Green, E. W., Fedele, G., Giorgini, F., & Kyriacou, C. P. (2014). A Drosophila RNAi collection is subject to dominant phenotypic effects. *Nature Methods*, *11*(3), 222–223. https://doi.org/10.1038/nmeth.2856

Kotzbauer, P. T., Truax, A. C., Trojanowski, J. Q., & Lee, V. M.-Y. (2005). Altered Neuronal Mitochondrial Coenzyme A Synthesis in Neurodegeneration with Brain Iron Accumulation Caused by Abnormal Processing, Stability, and Catalytic Activity of Mutant Pantothenate Kinase 2. *Journal of Neuroscience*. https://doi.org/10.1523/JNEUROSCI.4265-04.2005

Vissers, J. H. A., Manning, S. A., Kulkarni, A., & Harvey, K. F. (2016). A Drosophila RNAi library modulates Hippo pathway-dependent tissue growth. *Nature Communications*, *7*, 10368. https://doi.org/10.1038/ncomms10368

Fly genetics:

Commercially available stocks & stock sources:

- *UAS-dPANK/fbl-RNAi (P{KK109160}VIE-260B*; VDRC ID #101437*)*
- *UAS-dPPCDC/ppcdc-RNAi (P{KK109377}VIE-260B*; VDRC ID #104495*)*
- *UAS-mtacp-RNAi (y[1] v[1]; P{y[+t7.7] v[+t1.8]=TRiP.HM05206}attP2*, Bloomington #29528*)*
- *UAS-PDK-RNAi (y[1] v[1]; P{y[+t7.7] v[+t1.8]=TRiP.JF03050}attP2,* Bloomington #28635*) = PDK-RNAi Strain 1*
- *UAS-PDK-RNAi (y[1] sc[*] v[1]; P{y[+t7.7] v[+t1.8]=TRiP.GL00009}attP2,* Bloomington #35142*) = PDK-RNAi Strain 2*
- *UAS-SIRT4-RNAi (y[1] sc[*] v[1]; P{y[+t7.7] v[+t1.8]=TRiP.HMS00944}attP2,* Bloomington #33984*) = SIRT4-RNAi Strain 1*
- *UAS-SIRT4-RNAi (y[1] sc[*] v[1]; P{y[+t7.7] v[+t1.8]=TRiP.GL00548}attP2,* Bloomington #36588*) = SIRT4-RNAi Strain 2*
- *UAS-bsk-RNAi (y[1] sc[*] v[1]; P{y[+t7.7] v[+t1.8]=TRiP.GL00603}attP40,* Bloomington #36643*)*
- *UAS-EGFP (w[*]; P{w[+mC]=UAS-2xEGFP}AH2,* Bloomington #6874*) = "UAS-GFP"*
- *UAS-EYFP (y[*] w[*]; P{w[+mC]=UAS-2xEYFP}AH3,* Bloomington #6660*) = UAS-YFP"*
- *w1118 (w[1118],* Bloomington #3605*)*
- *Actin-GAL4 (y[1] w[*]; P{w[+mC]=Act5C-GAL4}25FO1/CyO, y[+]*, Bloomington #4414), ubiquitous driver
- *MS1096-GAL4* (*w[1118] P{w[+mW.hs]=GawB}Bx[MS1096]; P{w[+mC]=UAS-Dcr-2.D}2*, Bloomington #25706), driver expressed in the wing-pouch, used here with or without the *UAS-Dcr-2* element (see list below for stocks created for this project)

Stocks created for individual experiments, using the lines listed above:

- *UAS-dPANK/fbl-RNAi* (30B only, lines "F10" and "F20" established through mitotic recombination). For all experiments shown in this paper "F20" was used.
- *UAS-dPPCDC/ppcdc-RNAi* (30B only, lines "P7" and "P17" established through mitotic recombination). For all experiments shown in this paper "P7" was used.
- *Actin-GAL4::UAS-GFP/CyO*
- *Actin-GAL4::UAS-dPANK/fbl-RNAi F20/CyO* (30B only)
- *MS1096-GAL4; UAS-GFP*
- *MS1096-GAL4; UAS- dPANK/fbl-RNAi F20/CyO* (30B only)
- *MS1096-GAL4; UAS-PPCDC-RNAi F20/CyO* (30B only)

Detailed fly genotypes, sorted by figure:

Figure 3A:

- Control = *Act-GAL4 / UAS-GFP*
- dPANK-fbl-RNAi strain 1 = *Act-GAL4 / UAS-dPANK-fbl-RNAi "F10"*
- dPANK-fbl-RNAi strain 2 = *Act-GAL4 / UAS-dPANK-fbl-RNAi "F20"*

Figure 3B:

- Control = *Act-GAL4 / UAS-GFP*
- dPANK-fbl-RNAi = *Act-GAL4 / UAS-dPANK-fbl-RNAi "F20"*

Figure 4A:

- Control = *MS1096; UAS-GFP / UAS-GFP*

Figure 4B:

- Control + dcr2 = *MS1096; UAS-Dcr-2 / UAS-GFP*

Figure 4C:

- dPANK/fbl-RNAi = *MS1096; UAS-GFP / UAS-dPANK/fbl-RNAi "F20"*

Figure 4D:

- dPANK/fbl-RNAi + dcr2 = *MS1096; UAS-Dcr-2 / UAS-dPANK/fbl-RNAi "F20"*

Figure 4E:

- dPPCDC/ppcdc-RNAi = *MS1096; UAS-GFP / UAS-dPPCDC/ppcdc-RNAi "P7"*

Figure 4F:

- dPPCDC/ppcdc-RNAi + dcr2 = *MS1096; UAS-Dcr-2 / UAS-dPPCDC/ppcdc-RNAi "P7"*

Figure 4G:

- dPANK/fbl-RNAi + dPPCDC/ppcdc-RNAi = *MS1096; UAS-dPANK/fbl-RNAi "F20" / UAS-dPPCDC/ppcdc-RNAi "P7"*

Figure 4H:

- mtACP-RNAi = *MS1096; UAS-GFP / UAS-mtACP-RNAi (#29528)*

Figure 4I:

- mtACP-RNAi + dcr2 = *MS1096; UAS-Dcr-2 / UAS-mtACP-RNAi (#29528)*

Figure 5A:

- dPPCDC/ppcdc-RNAi = *MS1096; UAS-GFP / UAS-dPPCDC/ppcdc-RNAi "P7"*; +/- DCA in the fly food

Figure 5D:

- Control = *MS1096; UAS-GFP / UAS-dPPCDC/ppcdc-RNAi*

- RNAi knockdown: *MS1096; UAS-RNAi (PDK or SIRT4) / UAS-dPPCDC/ppcdc-RNAi "P7"*
- PDK-RNAi strain 1 = Bloomington #28635
- PDK-RNAi strain 2 = Bloomington #35142
- SIRT4-RNAi strain 1 = Bloomington #33984
- SIRT4-RNAi strain 2 = Bloomington #36588

Appendix Figure 1A:

- Control = w1118
- dPANK/fbl-RNAi (KK) = *UAS-dPANK/fbl-RNAi (KK) VDRC #101437*
- dPANK/fbl-RNAi strain 1 = *UAS-dPANK/fbl-RNAi "F10"*
- dPANK/fbl-RNAi strain 2 = *UAS-dPANK/fbl-RNAi "F20"*
- dPPCDC/ppcdc-RNAi (KK) = *UAS- dPPCDC/ppcdc-RNAi (KK) VDRC #104495*
- dPPCDC/ppcdc-RNAi strain 1 = *UAS- dPPCDC/ppcdc-RNAi "P7"*
- dPPCDC/ppcdc-RNAi strain 2 = *UAS- dPPCDC/ppcdc-RNAi "P17"*

Appendix Figure 1B:

- Control = *Act-GAL4 / UAS-GFP*
- dPANK/fbl-RNAi strain 1 = *Act-GAL4 / UAS-dPANK/fbl-RNAi "F10"*
- dPANK/fbl-RNAi strain 2 = *Act-GAL4 / UAS-dPANK/fbl-RNAi "F20"*

Appendix Figure 1C:

- Control = *Act-GAL4 / UAS-GFP*
- dPPCDC/ppcdc-RNAi strain 1 = *Act-GAL4 / UAS-dPPCDC/ppcdc-RNAi "P7"*
- dPPCDC/ppcdc-RNAi strain 2 = *Act-GAL4 / UAS-dPPCDC/ppcdc-RNAi "P17"*

Appendix Figure 2:

Crosses and resulting offspring are being presented in the figure itself.

UAS-fbl-RNAi used: *UAS-dPANK/fbl-RNAi "F20"*

Appendix Figure 3B:

- Control + GFP = *MS1096; UAS-GFP / UAS-GFP*

Appendix Figure 3C:

- dPANK/fbl-RNAi + GFP = *MS1096; UAS-GFP / UAS-dPANK/fbl-RNAi "F20"*

Appendix Figure 3D:

- Control + GFP = *MS1096; UAS-GFP / UAS-GFP*

Appendix Figure 3E:

- mtACP-RNAi + GFP = *MS1096; UAS-GFP / UAS-mtACP-RNAi (#29528)*

Appendix Figure 4A:

- dPPCDC/ppcdc-RNAi + UAS-GFP = *MS1096; dPPCDC/ppcdc-RNAi / UAS-GFP*

Appendix Figure 4B:

- dPPCDC/ppcdc-RNAi + UAS-YFP = *MS1096; dPPCDC/ppcdc-RNAi / UAS-YFP*

Appendix Figure 4C:

- dPPCDC/ppcdc-RNAi + UAS-RNAi #36643 = *MS1096; dPPCDC/ppcdc-RNAi / UAS-Bsk-RNAi (#36643)*

Appendix Figure 5A-E:

- Control = *MS1096; UAS-GFP / UAS-GFP*
- PDK-RNAi strain 1 = *MS1096; UAS-GFP / UAS-PDK-RNAi #28635*
- PDK-RNAi strain 2 = *MS1096; UAS-GFP / UAS-PDK-RNAi #35142*
- SIRT4-RNAi strain 1 = *MS1096; UAS-GFP / UAS-SIRT4-RNAi #33984*
- SIRT4-RNAi strain 2 = *MS1096; UAS-GFP / UAS-SIRT4-RNAi #36588*

Appendix Figure 5F:

- Control = *Act-GAL4 / UAS-GFP*
- PDK-RNAi strain 1 = *Act-GAL4 / UAS-PDK-RNAi #28635*
- PDK-RNAi strain 2 = *Act-GAL4 / UAS-PDK-RNAi #35142*

Appendix Figure 5G:

- Control = *Act-GAL4 / UAS-GFP*
- SIRT4-RNAi strain 1 = *Act-GAL4 / UAS- SIRT4-RNAi #33984*
- SIRT4-RNAi strain 2 = *Act-GAL4 / UAS- SIRT4-RNAi #36588*

Name	Vector	Promoter	Color
piSMART hEF1a/ TurboGFP (NTC)	pSMART	hEF1a	TurboGFP
	**Selection**	**Target**	**Target sequence**
	puromycine	Non-Targeting Control	Undisclosed
	**Source Clone ID**	**Cat.#**	
	SVC17010402	VSC11653	

Name	Vector	Promoter	Color
piSMART hEF1a/ TurboGFP (PANK2.1)	pSMART	hEF1a	TurboGFP
	**Selection**	**Target**	**Target sequence**
	puromycine	hPANK2	GCATATGCTTTGGATTATT
	**Source Clone ID**	**Cat.#**	
	V3IHSHEG_4828861	V3SH11252-224891211	

Name	Vector	Promoter	Color
piSMART hEF1a/ TurboGFP (PANK2.2)	pSMART	hEF1a	TurboGFP
	**Selection**	**Target**	**Target sequence**
	puromycine	hPANK2	GGAGGGGACTATGAGAGGT
	**Source Clone ID**	**Cat.#**	
	V3IHSHEG_8137144	V3SH11252-228199494	

Name	Vector	Promoter	Color
piSMART hEF1a/ TurboGFP (PANK2.3)	pSMART	hEF1a	TurboGFP
	**Selection**	**Target**	**Target sequence**
	puromycine	hPANK2	GGATTCAATGGACGGTCAC
	**Source Clone ID**	**Cat.#**	
	V3IHSHEG_9580135	V3SH11252-229642485	

Cell Lines	Company	Catalog Number
HEK293T	ATCC	CRL-3216
HEK293T NTC	this paper	
HEK293T PANK 2.1	this paper	
HEK293T PANK 2.1	this paper	
SH-SY-5Y	ATCC	CRL-2266
SH-SY-5Y NTC	this paper	
SH-SY-5Y PANK 2.1	this paper	
SH-SY-5Y PANK 2.3	this paper	
Schneider’s Drosophila Line 2 (“S2” cells)	ATCC	CRL-1963

Appedix Table 1: Vectors and Cell Lines

q-PCR target (human)	Forward primer	Reverse primer
hPANK1	TTCAGATGGGCAGCGAGAAG	AAGCAGGCCCTGAATCAGAC
hPANK2	GGGATCGACTGGGCTCTTAC	CCAGCTTGACCAGAGTTCCA
hPANK3	ATTGCAGACGGTGCTATGTG	CTCGGCTTGTCCATTGAAAC
hPANK4	GGTACACGAAGGCCTCATGG	AGAAGCTGCGGCTGAAAGTC
hGAPDH	ACCCACTCCTCCACCTTTG	CACCACCCTGTTGCTGTAG
hRPL27	CCGGACGCAAAGCTGTCATCG	CTTGCCCATGGCAGCTGTCAC
hHPRT	TGCTGACCTGCTGGATTAC	GCGACCTTGACCATCTTTG
hB2M	GGGATCGAGACATGTAAGC	GAGACAGCACTCAAAGTAG
hUBC	AGTCCCTTCTCGGCGATTC	TGGTGTCACTGGGCTCAAC

q-PCR target (*Drosophila*)	Forward primer	Reverse primer
dPANK/fumble	CCCGATTGCAACATCTGTCGG	CCAGCTTGGTGAGGGTTCC
dPPCDC/ppcdc	CCTCCTCTACGCCAGTTGTC	TTCCGGTGGCCGCTATCATC
RP49	CCGCTTCAAGGGACAGTATC	GACAATCTCCTTGCGCTTC
PDK	AATTTCTGACCAGGGAGGCG	CGTCGGACAGAGCCTTTAGA
SIRT4	TTCCAGAGCATACTGGCGTC	ACCGGTAGCTTGAGGTCCTT

Appendix Table 2 - qPCR Primers

Antibody	Source/Reference	Identifiers	Additional info.
mtACP (NDUFAB1, Rabbit polyclonal)	ThermoFisher Scientific	PA5-22191	WB (1:1000)
mtACP (NDUFAB1, Rabbit polyclonal)	ThermoFisher Scientific	PA5-22191	IF (1:500)
Lipoic Acid (Rabbit polyclonal)	Merck Millipore	437695	WB (1:1000)
PDH-E2 (Rabbit polyclonal)	Abcam	ab66511	WB (1:1000)
α-Tubulin (Mouse monoclonal, Clone B-5-1-2)	Sigma Aldrich	T5168	WB (1:5000)
GAPDH (Mouse monoclonal)	Fitzgerald	10R-G109A	WB (1:10000)
dPank/Fbl (Rabbit polyclonal)	PMID: 18407920		WB (1:1000)
hPANK2 (Rabbit polyclonal)	Sigma Aldrich	HPA 008440	WB (1:250)
hPANK2 (Mouse monoclonal)	Sigma Aldrich	WH 0080025M1	WB (1:300)
hPANK2 (Mouse monoclonal)	Gift from P. Kotzbauer		WB (1:1000)
hPANK2 (Mouse monoclonal, Clone OTI3H9)	Origene	TA5011321	WB (1:500)
hPANK2 (Rabbit polyclonal)	Gift from S. Jackowski		WB (1:2000)

Appendix Table 3 - Antibodies
