# Supplementary material for: CoA‐dependent activation of mitochondrial acyl carrier protein links four neurodegenerative diseases
Source: EMBO Mol Med. 2019 Nov 7;11(12):e10488. doi: 10.15252/emmm.201910488 (PMC6895606; doi:10.15252/emmm.201910488)
Supplement: Supplementary file 3 — Source Data for Figure 3 [file EMMM-11-e10488-s002.pdf]

Source Data for Figure 3

Original blots used for Figures 3B-C

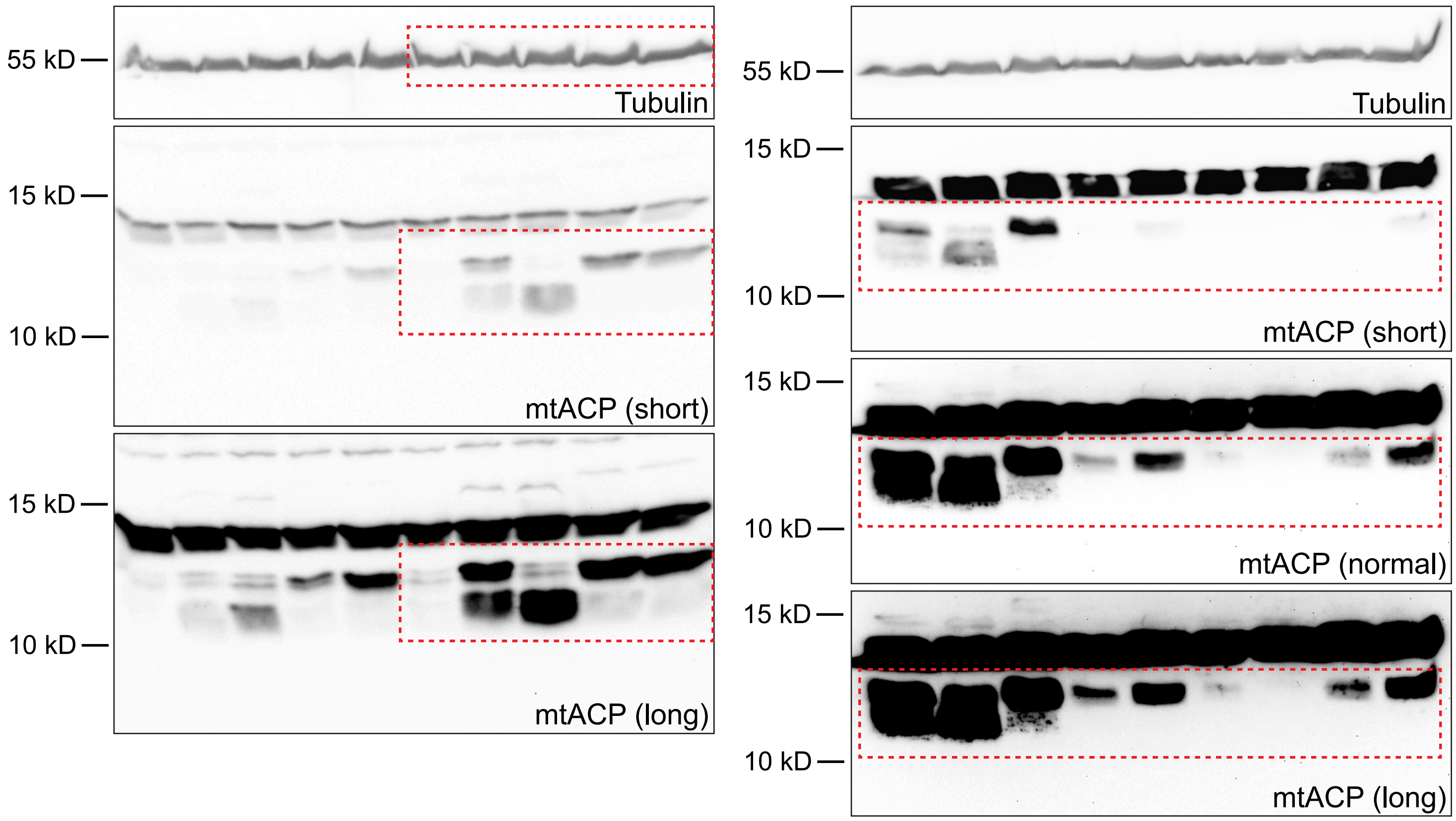

Source data for Figure 3: Full gel images for Western blots presented in Figure 3

(A) Full gel images for the anti-mtACP/Tubulin Western blot presented in Fig. 3B. For anti-mtACP two different exposure times were used to detect the different mtACP expressing constructs as well as endogenous mtACP. The part of the Western used for the main figure is outlined by a dashed line.

(B) Full gel images for the anti-mtACP/Tubulin Western blot presented in Fig. 3C. For anti-mtACP three different exposure times are presented. The whole Western blot was used for Fig 3D.
