# Supplementary material for: CoA‐dependent activation of mitochondrial acyl carrier protein links four neurodegenerative diseases
Source: EMBO Mol Med. 2019 Nov 7;11(12):e10488. doi: 10.15252/emmm.201910488 (PMC6895606; doi:10.15252/emmm.201910488)
Supplement: Supplementary file 4 — Source Data for Figure 4 [file EMMM-11-e10488-s003.pdf]

Source Data for Figure 4

Original blots used for Figures 4A

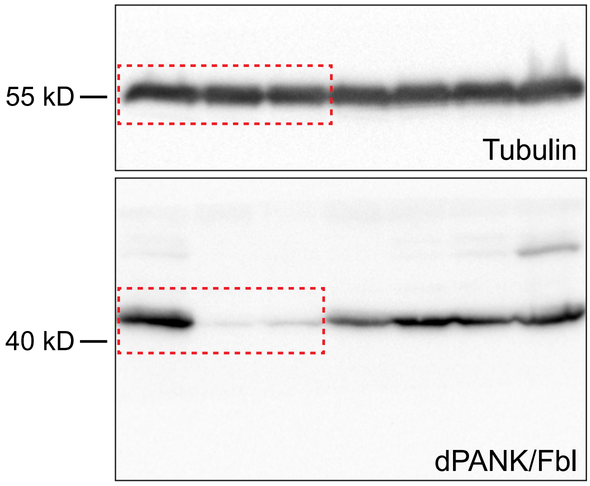

Source data for Figure 4: Full gel images for Western blots presented in Figure 4A
Original images for the anti-dPANK/fbl / Tubulin Western blot presented in Figure 4A. Red dashed squares outline the parts used to assemble the Western in the main figure.
