# Supplementary material for: CoA‐dependent activation of mitochondrial acyl carrier protein links four neurodegenerative diseases
Source: EMBO Mol Med. 2019 Nov 7;11(12):e10488. doi: 10.15252/emmm.201910488 (PMC6895606; doi:10.15252/emmm.201910488)
Supplement: Supplementary file 5 — Source Data for Figure 7 [file EMMM-11-e10488-s004.pdf]

Source Data for Figure 7

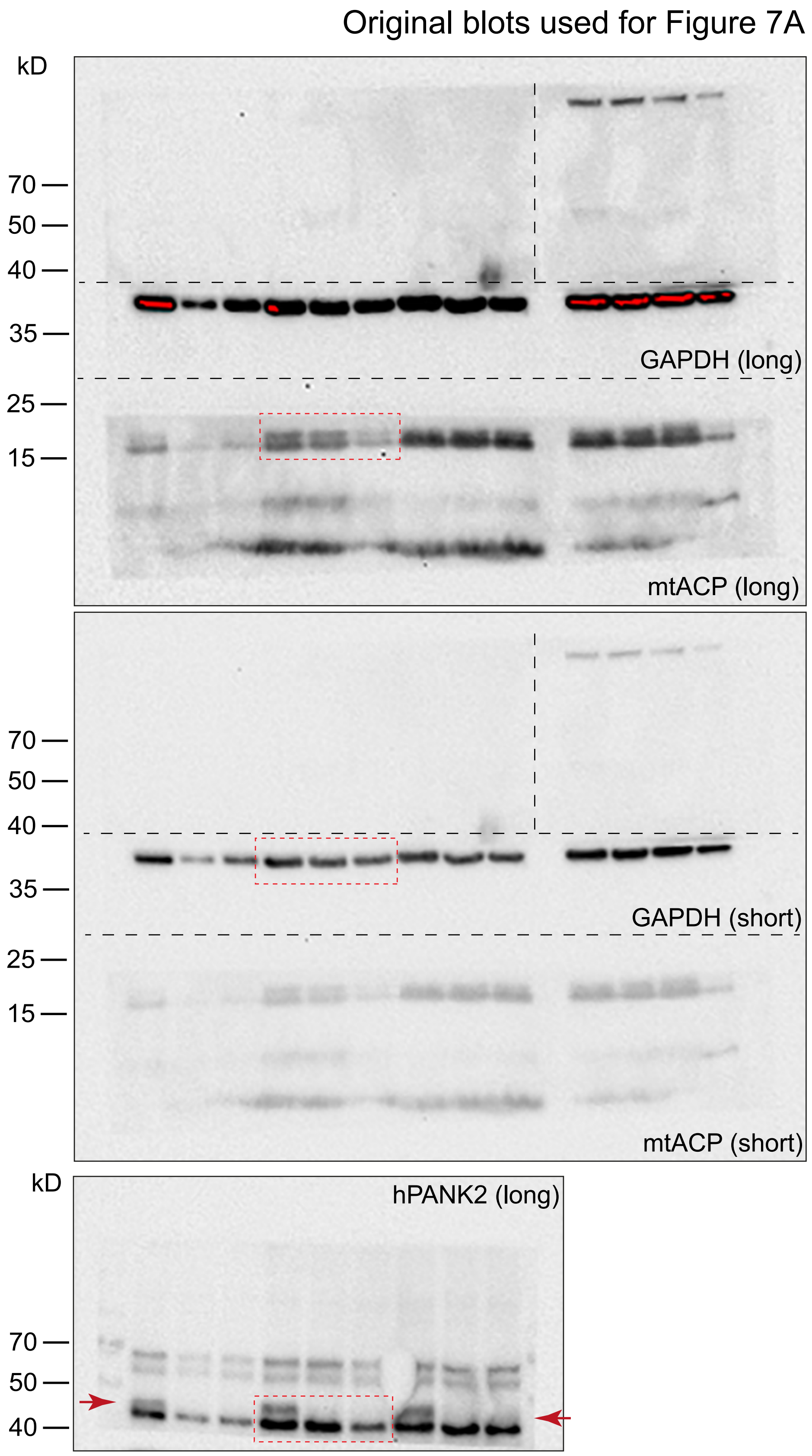

Source data for Figure 7A: Full gel images for Western blots presented in Figure 7A (HEK293T)

Original images for the anti-hPANK2/mtACP/GAPDH Western blots presented in Fig. 7A. The Western samples were run on the same gel, blotted and the membrane then cut into smaller pieces (according to the expected protein size), which were probed for mtACP and GAPDH separately. Black dashed lines indicate the individual blots, lying next to each other. A separate Western blot was run to detect hPANK2. A longer exposure time was needed to visualise hPANK2 and mtACP, while a shorter one was used for the control GAPDH. Red dashed squares outline the parts used to assemble the Western in Figure 7A.

Source Data for Figure 7

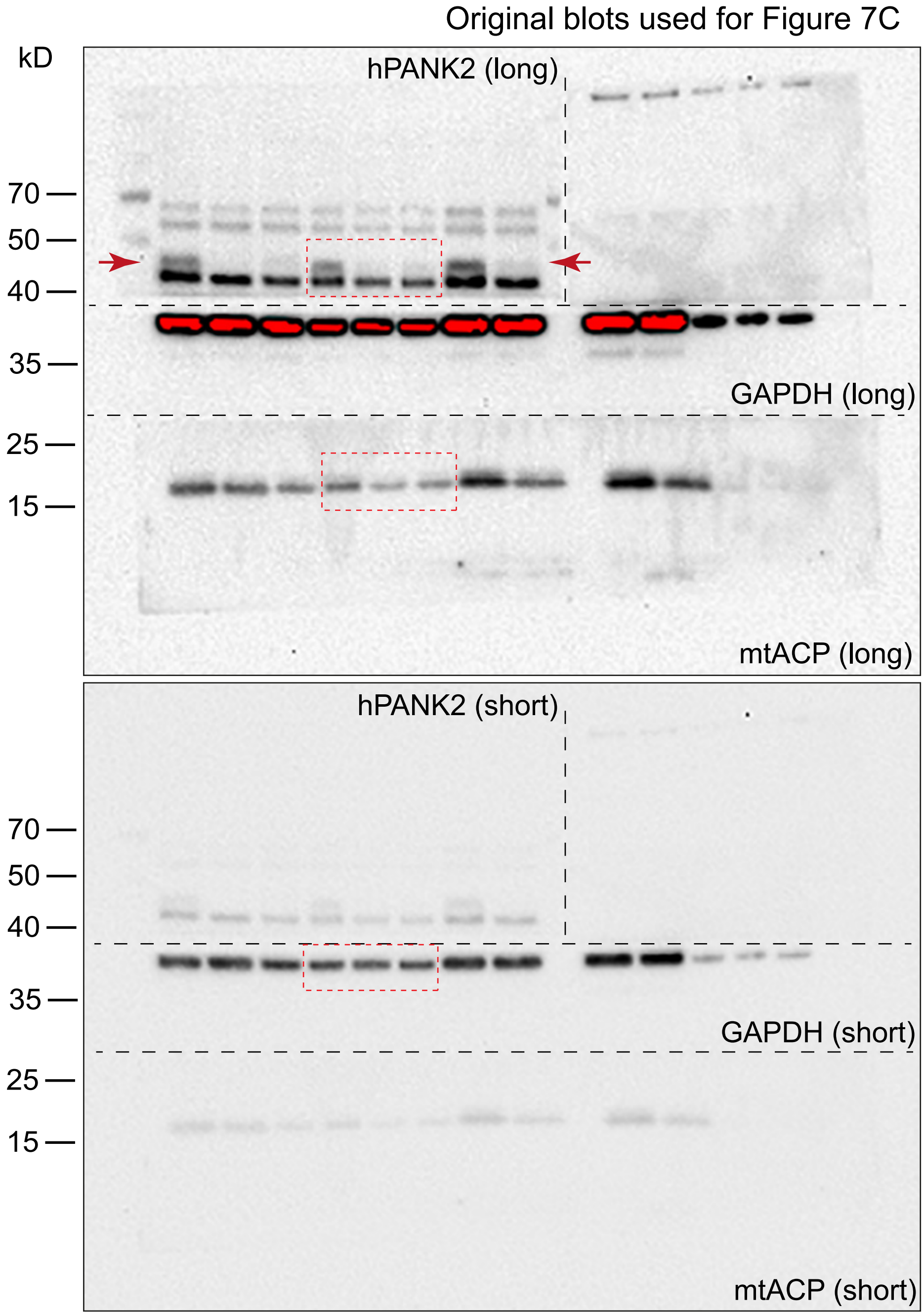

Source data for Figure 7C: Full gel images for Western blots presented in Figure 7C (SH-SY-5Y cells)

Original images for the anti-hPANK2/mtACP/GAPDH Western blot presented in Fig. 7C. The Western samples were run on the same gel, blotted and the membrane then cut into smaller pieces (according to the expected protein size), which were probed for the three antibodies separately. Black dashed lines indicate the individual blots, lying next to each other. A longer exposure time was needed to visualise hPANK2 and mtACP, while a shorter one was used for the control GAPDH. Red dashed squares outline the parts used to assemble the Western in Figure 7C.
